# Two-Dimensional and Three-Dimensional Changes in Deformational Head Shapes During Repositioning Therapy and Cranial Remolding Treatment

**DOI:** 10.3390/jcm13247689

**Published:** 2024-12-17

**Authors:** Tiffany Graham, Jijia Wang, Fabian A. Calderon, Victoria Moses, Rami R. Hallac

**Affiliations:** 1 Department of Prosthetics-Orthotics, University of Texas Southwestern Medical Center, 6011 Harry Hines Blvd, Dallas, TX 75390, USA; 2 Department of Applied Clinical Research, University of Texas Southwestern Medical Center, 6011 Harry Hines Blvd, Dallas, TX 75390, USA; 3 Analytical Imaging and Modeling Center, Children’s Health, 1935 Medical District Drive, Dallas, TX 75235, USA

**Keywords:** plagiocephaly, brachycephaly, cranial vault asymmetry, cranial vault asymmetry index, cephalic index, cranial proportion, cranial ratio, cranial remolding orthosis, helmet, repositioning therapy

## Abstract

**Backgrounds/Objectives:** The surge in deformational head shapes (DHSs) over the past 30 years has led to increased interest in comparing the treatment options of Repositioning Therapy (RT) and a Cranial Remolding Orthosis (CRO). This study investigates the amount and rate of 2D and 3D correction in infants with DHSs during these treatments. **Methods:** A total of 34 infants with DHSs were enrolled (RT group, *n* = 18; CRO group, *n* = 16). Infants were discharged after achieving correction or reaching 12 months of age. Two-dimensional scan/caliper measurements and three-dimensional scan measurements were collected at treatment initiation and conclusion (or 12 months of age). **Results:** Asymmetric infants in the RT group averaged a 2dCVAI reduction of 3.59 ± 1.57 and 3dCVAI correction of 12.17 ± 13.02 versus 4.44 ± 2.99 and 21.72 ± 15.36 correction in the CRO group (2d *p* = 0.6656; 3d *p* = 0.1417). Disproportionate infants in the RT group averaged a 2dCI reduction of 3.13% ± 2.57% and 3dCI reduction of 24.53 ± 24.01 while the CRO group averaged 5.21% ± 2.78% and 55.98 ± 25.77 (2d *p* = 0.0383*; 3d *p* = 0.0254*). Asymmetrical RT mean 2dCVAI weekly change was 0.21 ± 0.15 while CRO was 0.23 ± 0.17 (*p* = 0.7796). The 3dCVAI weekly change was 1.05 ± 1.55 in the RT group versus 1.17 ± 0.95 in the CRO group (*p* = 0.4328). Disproportionate RT mean 2dCI weekly change was 0.12 ± 0.11 while CRO was 0.23 ± 0.11 (*p* = 0.0440*). The 3dCI weekly change was 0.87 ± 0.91 in the RT group versus 3.02 ± 2.16 in the CRO group (*p* = 0.0143*). **Conclusions:** Results indicate that CRO treatment achieves greater total correction and rate of correction. Statistical significance was found in the treatment of disproportional DHSs, but further investigation is needed with a larger sample size.

## 1. Introduction

**Figure 1:**
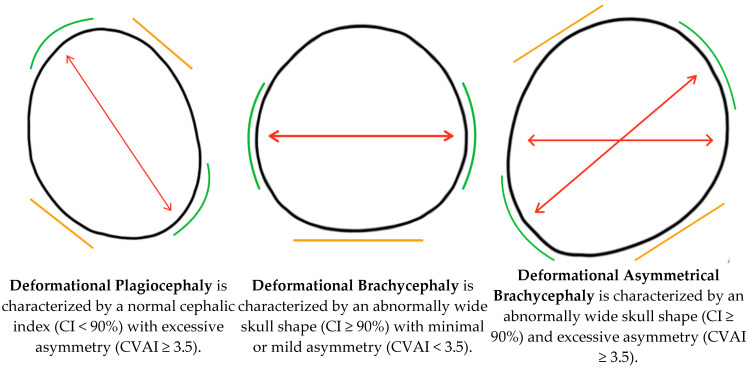
A pictorial representation of a superior view of the deformational head shapes that are of focus in this study, along with a description of their general presentations. The orange lines represent flattened areas, the green lines represent bossed areas, and the red arrows represent the areas where cranial growth is being driven into.

Since the initiation of the American Academy of Pediatrics (AAP) “Back to Sleep Campaign” in 1992, there has been a significant increase in the incidence of deformational head shapes (DHSs) [1]. The AAP’s campaign recommended that sleeping infants should be placed in a supine position to decrease the likelihood of sudden infant death syndrome (SIDS). Despite the success in reducing the incidence of SIDS, asymmetric cranial deformations have risen sharply, with an estimated 1 per 60 births, as opposed to 1 per 300 births before AAP’s recommendation [2]. Disproportional cranial deformations have also followed this trend as sleeping supine predisposes infants to external forces on the occiput, resulting in a flattening of an infant’s soft and malleable cranium [3]. The DHSs of focus in this study include deformational plagiocephaly (DP), deformational brachycephaly (DB), and deformational asymmetrical brachycephaly (DAB) (Figure 1). Figure 1 visually displays each of the DHSs by demonstrating where growth is being redirected, the bossed areas, and the flattened areas, and includes a description of how these head shapes are characterized and what cranial measurements they need to be classified as such. DP is an asymmetrical skull deformity in which flattening occurs on one side of the head and is often characterized as “oblique” [3,4]. A major risk factor of DP is congenital muscular torticollis, which causes an infant’s head to twist and turn to one side due to shortened or tight sternocleidomastoid muscles, resulting in a positional preference for one side [5]. Infants with congenital muscular torticollis (CMT) should be referred immediately to physical therapy (PT) to stretch the tightened sternocleidomastoid and achieve full range of motion of the neck. DB is defined as a symmetrical flattening of the occiput, resulting in a wider-than-normal head shape [6]. DAB is a combination of DP and DB and is classified as an asymmetric and wider head shape.

Cranial measurements track the progression of DHSs and how they respond to treatment. The cranial measurements utilized in this study include the Cephalic Index (CI) and the Cranial Vault Asymmetry Index (CVAI) and describe disproportionality and asymmetry, respectively. Since the Back to Sleep Campaign, the normal CI range has increased to 82–86%, and the value is calculated by dividing the cranial width by the cranial length and then multiplying the results by 100 [7]. CVAI assesses the diagonals of the head 30° from the midline, and normal values are described as those < 3.5 [8,9]. CVAI is computed by subtracting the lesser diagonal from the greater diagonal, dividing the result by the greater diagonal, and then multiplying the quotient by 100 [9].

This study will focus on the two most common treatment modalities for DHSs, repositioning therapy (RT) and cranial remolding orthosis (CRO) therapy, and their effects on two-dimensional (2D) and three-dimensional (3D) correction. RT is a positional strategy that keeps infants off the flat side of their heads and increases the time spent on the opposite or bossed side of their heads. RT strategies include spending more time in a prone position, or “tummy time”, and spending less time in car seats, rockers, or bouncers [10]. For infants with asymmetry, RT also focuses on directing their attention to their non-preferred side. CROs are classified as Class II medical devices by the Food and Drug Administration (FDA) and are intended to redirect cranial growth into the desired head shape [11]. Desired growth patterns are accomplished by the orthosis fitting snugly over the prominent areas of the cranium while leaving room over the flattened areas to redirect cranial growth into [12]. CROs may be fabricated by casting a patient’s head with a plaster bandage or by using a scan to create a positive model of the infant’s cranium. The cast or scan is then modified (i.e., symmetry increased and/or proportion reduced) based on the infant’s clinical presentation, and plastic is traditionally thermoformed over the modified model to generate a custom-made orthosis [12]. CROs may also be created utilizing 3D printing technology. The general clinical guidelines for CRO wear time compliance is 22–23 h per day, with overall treatment lasting anywhere between 3 and 6 months [10]. RT is considered effective as the sole treatment for DHSs in mild to moderate presentations, while CRO treatment is recommended for infants with moderate to severe presentations following unsuccessful conservative treatment [13,14].

The debate comparing the effectiveness of 2D RT and CRO treatments has been investigated by several researchers. The Congress of Neurological Surgeons recommended with high clinical certainty that “repositioning is inferior to physical therapy and to use of a helmet, respectively” [15]. JM Graham et al. compared the two treatment modalities in treating asymmetric deformations and found that CRO treatment was not only more effective in decreasing diagonal differences (DD) when compared to RT but also resulted in infants having a mean final DD closer to the mean DD in infants with a normal head shape [16]. Shargo et al. found that when treating DP, CROs were superior to RT as infants who underwent CRO treatment experienced a more significant improvement in cranial vault asymmetry (CVA) and had more notable improvement in severe presentations [17]. Naidoo et al. assessed asymmetrical and proportional head shapes and found, with statistical significance, a better improvement in CI and Cranial Vault Asymmetry (CVA) when using a CRO compared to RT, indicating that “infants have greater normalization in head shape when a helmet is used versus repositioning” [10]. Most of the literature demonstrates that CROs more effectively correct 2D DHSs compared to RT.

Traditionally, cranial evaluations have been performed with 2D measurements, but these measurements do not always accurately portray an infant’s 3D head shape [7,18]. Although 3D measurements are rarely used to assess an infant’s head shape, there is growing interest in this area with the availability of 3D scanning technology [7,18]. In 2019, Kunz et al. conducted a 3D investigation into the comparison of CRO and RT treatments and demonstrated that CRO therapy led to “significantly better long-term outcomes compared with active repositioning of physiotherapy alone” [19]. More recently, Kajita et al. found that, like 2D correction, CRO treatment provided significant improvements in 3D outcomes and that the infants who achieved the greatest improvement were younger and had more severe initial presentations [18]. The purpose of this study is to compare RT and CRO treatment for DHSs, not only in 2D but 3D as well, within a single treatment center and determine if one of these treatments is more effective in treating DHSs than the other by 12 months of age.

## 2. Materials and Methods

The study design is a prospective study with data collection spanning from 1 February 2019 to 30 August 2022. Two-month-old infants were recruited by treating orthotists at the University of Southwestern Medical Center’s Prosthetic–Orthotic Clinic if an infant met the inclusion criteria relevant to this study and was referred to the clinic for treatment of a deformational head shape. Efforts were made to ensure that infants were enrolled in the study as soon after their referral as possible. This was done to increase the likelihood of successful RT or CRO treatment. Age at treatment initiation is a major factor in CRO treatment outcomes, as a younger initiation age is correlated with a greater rate of correction, and the greatest correction will occur at four to six months of age and will quickly decline with increased age [20]. Likewise, an earlier age for RT is associated with better outcomes as by four to six months of age, infants develop greater strength and control of their heads, thus making it harder to keep an infant in the desired position [21]. Another important factor in the treatment of DHSs is prematurity, as gestational age is a better predictor for how long an infant stays in treatment as opposed to birth age [22]. In this study, the age of a premature infant was adjusted by subtracting the number of weeks of prematurity from a child’s birth age. Infants born at or after 38 weeks of gestation were considered full-term, and if an infant was born prematurely, they would have their age adjusted to a 40-week gestation period. For example, an infant born prematurely at 34 weeks of gestation would have six weeks subtracted from their birth age, and the result would be their adjusted age.

Institution Review Board approval was obtained from the University of Texas Southwestern Medical Center (Protocol #STU 032017-036), and all infants were treated at the University of Texas Southwestern Medical Center’s Prosthetic–Orthotic Clinic. Informed consent was obtained from the caregivers of participating infants; they were educated on the goals and inclusion/exclusion criteria of the study. Caregivers did not receive financial compensation for their participation but had their treatment fully covered by the funding received from the AOPA Research Award, which was granted by the Center for Orthotics and Prosthetics Learning and Outcomes/Evidence-based Practice. This funding covered all appointments, which included the evaluation, repositioning instructions (for those in the RT group), the CRO (for those in the CRO group), and all follow-up visits with the orthotist. PT was also covered for participants if they were diagnosed with CMT, with subjects undergoing therapy concurrently with their DHS treatment. By having all aspects of treatment covered, the likelihood of extraneous variables, such as bias resulting from treatment costs and the influence of untreated CMT, was reduced.

To be included in this study, subjects had to be diagnosed with a DHS of interest to the study and had to have 2D cranial measurements (CI and CVAI) that were in accordance with their diagnosis. The necessary CI and CVAI values for each DHS were as follows: DP: CVAI ≥ 3.5 and CI < 90%; DB: CVAI < 3.5 and CI ≥ 90%; DAB: CVAI ≥ 3.5 and CI ≥ 90%. These cranial measurements were acquired using caliper measurements, which may have slightly differed from the information acquired from an infant’s scan. Two-dimensional cranial measurements were collected using calipers and the Smartsoc or STARscanner scanning systems and the use of Measurement and Comparison Utility or Cranial Comparison Utility, respectively (Orthomerica Products, Orlando, FL and Vorum Research Corporation, Vancouver, BC, Canada). Based on the caliper measurements acquired by the evaluating orthotist, infants were stratified into their respective severity groups. To classify the severity of a DHS, the Children’s Healthcare of Atlanta (CHOA) severity scale was utilized to describe the degree of asymmetry an infant presented with based on their CVAI while parameters were set in the study regarding CI and disproportionality (Table 1).

Table 1 demonstrates the asymmetric and disproportional cranial measurements needed for classification at a certain level of severity and range from ‘mild’ to ‘very severe’. Mild asymmetry was characterized as CVAI ≥ 3.5 and less than 6.5, while mild disproportion was defined as less than 90%. Moderate asymmetric deformations were classified by a CVAI greater than or equal to 6.5 and less than 8.75, while a moderate proportional deformation was classified by a CI ≥ 90% and less than 95%. An infant was classified as having a severe asymmetric deformation when their CVAI was ≥ 8.75 but less than 11, and a severe proportional deformation was characterized by a CI ≥ 95% but less than 100%. Very severe asymmetrical and disproportional presentations were classified as CVAI ≥ 11 and CI ≥ 100%, respectively. To classify the severity of infants with DAB, the greater of the two severity classifications was chosen based on their CVAI and CI (i.e., an infant with a mild CVAI and severe CI would be classified as severe DAB). The rationale for appropriately classifying infants by their DHS severity is that severity is an important contributing factor to the success of CRO treatment. Infants with a less severe presentation will have greater success with CRO treatment, and the severity of an infant’s presentation is a more important predictor of treatment outcomes than age at initiation [12,23].

All RT infants started treatment at two to three months of age. Caregivers received standardized instructions on how to achieve the desired positions that were specific to an infant’s DHS diagnosis. Infants undergoing RT were seen monthly by the treating orthotist until the DHS resolved (or they were 12 months of age), and 2D measurements were collected using calipers and a scanner. Infants in the CRO group were enrolled at four to six months of age, at which time a scan of the infant’s head was taken to be used to fabricate a STARband side-opening CRO, which took approximately two weeks to fabricate. Infants were seen every one to three weeks for follow-up appointments and potential adjustments of the device and received a scan by the orthotist every six to eight weeks. Infants continued this timeline until their head shape was corrected or they reached 12 months of age (end of study participation). The way subjects were divided into these two treatment groups was based on caregiver preference. All enrolled infants began treatment in the RT group. Caregivers were then given the choice to switch to the CRO group at either their four-, five-, or six-month follow-up visit if the DHS had not yet resolved with RT treatment. If caregivers chose not to enroll in the CRO group, they continued with RT for the rest of the study and were part of the RT treatment group. If caregivers did choose to enroll in CRO treatment, all measurements collected during their RT were disregarded and their CRO evaluation appointment was considered the start of their treatment and measurements collected past this point were included in data analysis.

Infants were considered to have normalized (corrected) cranial measurements if their cranial asymmetry (i.e., diagonal difference) was less than 6 mm and 2dCI was less than 90% and if they were “visually corrected”. Visual correction was determined in reference to Argenta’s Classifications, which is a scale based on cranial shape and secondary deformation to determine an infant’s specific DHS and if correction has been achieved [24]. Based on these classifications, DB was corrected if the deformation was not clinically apparent or was Type I, and DP was corrected if the deformation was not clinically apparent or was Type I or Type II [25].

Three-dimensional measurements were acquired using the 3dMD system (3dMD, Atlanta, GA, USA), a non-ionizing scanning system. Data analysis was performed using custom-designed programs written in MATLAB version R2024a (MathWorks, Natick, MA, USA). A 3D template with symmetrical mesh connectivity was developed from a healthy subject’s scan. Each patient’s 3D image was aligned to this template using rigid translation and rotation, guided by 25 anatomical landmarks. An additional 40 landmarks were automatically placed on the head’s surface using radial lines from the image center. The template was then scaled and deformed to fit each scan through a thin-plate spline algorithm and closest point deformation, maintaining consistent polygonal indexing for all images [26,27,28].

**Figure 2:**
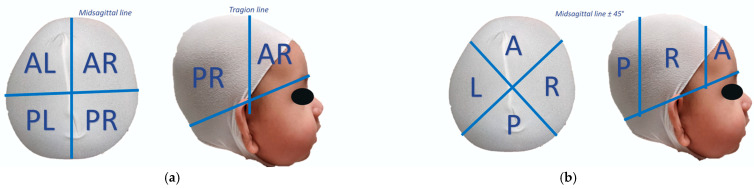
A visual representation of the division of an infant’s head into quadrants for calculation of their 3D measurements. (**a**) Demonstrates the quadrants used to calculate 3dCVAI with the following definitions: *AL*: anterior left; *AR*: anterior right; *PL*: posterior left; *PR*: posterior right. (**b**) Demonstrates the quadrants used to calculate 3dCI with the following definitions: *A*: anterior; *P*: posterior; *L*: left; *R*: right. The midsagittal and tragion lines are used to divide the quadrants in (**a**), while diagonals 45° from the midsagittal line were used to divide the quadrants in (**b**).

3D scans were acquired at the beginning and end of the treatment modality or when an infant reached 12 months of age. To assess an infant’s 3D asymmetry, 3D images were divided into anterior and posterior quadrants, each with left and right quadrants, and were used to calculate 3dCVAI. The net percent change in asymmetry (3dCVAI) was calculated as the net mean radial growth in the flattened quadrants minus the net mean radial growth in the bossed quadrants, with the difference being divided by the net mean radial growth in the bossed quadrants and the result being multiplied by 100. The 3D proportional changes were assessed by dividing 3D images into anterior, posterior, left, and right quadrants, with each of the quadrants being separated by a vertical plane 45 degrees from the midsagittal line. The net percent change in length growth (or 3dCI) was calculated as the net mean radial growth in the anterior and posterior quadrants minus the net mean radial growth in the left and right quadrants divided by the net mean radial anterior and posterior growth and the result was multiplied by 100. The division of quadrants used for calculating 3dCVAI and 3dCI are displayed in Figure 2. Figure 2a displays an infant’s head divided into four quadrants using the midsagittal and tragion lines: anterior left, anterior right, posterior left, and posterior right. Figure 2b displays an infant’s head divided into four quadrants using two midsagittal lines at 45° angles to create the following quadrants: anterior, posterior, left, and right. These quadrants are used in calculating 3dCVAI and 3dCI; the formulas are presented in Table 2. Table 2 contains the formulas for calculating 2dCVAI, 2dCI, 3dCVAI, and 3dCI. The weekly rate of change in each of the 2D and 3D measurements (i.e., 2dCVAI, 2dCI, 3dCVAI, and 3dCI) was calculated by dividing each measurement by the total number of weeks an infant was actively in treatment.

The demographics and clinical measurements collected for all infants were organized into an Excel document to be used in statistical analyses. Infants were not included in the data analyses if they did not receive their final 3dMD scan or if they were lost to follow-up during the study. Infants were grouped into two groups, asymmetrical and disproportional, based on their diagnosed DHS. Subjects diagnosed with DP and DAB were part of the asymmetrical group since these subjects have asymmetrical head shapes, and the target of their treatment is to reduce asymmetry. Subjects with disproportional head shapes, DB and DAB, were part of the disproportional group since the target of treatment in these individuals is to reduce disproportion. The 2D and 3D statistical analyses were performed to compare RT and CRO treatment outcomes as well as patient demographics between the asymmetrical and disproportional groups. Fisher’s exact test was performed to determine if statistical significance existed in the gender distribution between the two treatment modalities. A Mann–Whitney U test was performed to test statistical significance in starting ages between the two treatment groups. To compare the duration of treatment between 2D and 3D treatment, a Wilcoxon signed-rank test was carried out. Each of the 2D and 3D treatment measurements was compared with a Mann–Whitney U test to determine if statistical significance existed between RT and CRO regarding the rate of correction achieved. These rates of change included 2dCVAI, 2dCI, 3dCVAI, and 3dCI. A Mann–Whitney U test was performed to test for statistical significance in 2D treatment duration alone between the RT and CRO groups. The level of statistical significance was set at 5%. Effect sizes were calculated as appropriate. Subject information and data collection were organized in a Microsoft Excel spreadsheet, and all the statistical analyses were conducted using SAS 9.4 (SAS Institute Inc., Cary, NC, USA).

## 3. Results

With the inclusion criteria, 34 infants (20 male and 14 female) were enrolled in the study. Of the 34 enrolled infants, 12 were part of the DP group, eight were part of the DB group, and 14 were part of the DAB group. Based on these head shapes, the infants were then reorganized into asymmetrical and disproportional groups. This resulted in 26 infants in the asymmetrical group (16 male and 10 female), of whom 12 were in the RT group and 14 were in the CRO group. In the disproportional group, 22 infants were included (11 male and 11 female), with 12 being part of the RT group and 10 being in the CRO group. The reason for the asymmetrical and disproportional groups having more combined infants than the number of subjects who participated in the study is due to subjects diagnosed with DAB. Since DAB is an asymmetrical and disproportional head shape, both presentations will be the target of treatment, and thus, their inclusion in both groups is necessitated. These demographics, 2D treatment outcomes, and 2D statistical analyses can be seen in Table 3. The demographics in Table 3 include the corrected initiation age and gender distribution, while the 2D treatment outcomes include initial and final 2dCVAI and 2dCI measurements, the total and rate of 2dCVAI and 2dCI correction achieved, and the duration of 2D treatment. For each of the demographics, means and standard deviations are displayed in addition to the comparison *p*-values and effect sizes for the statistical analyses performed to compare RT to CRO.

The timeframes for starting and ending 2D treatment were assessed. When looking at the age of treatment initiation, corrected for prematurity, on average, RT infants in the asymmetrical group began treatment at 9.67 weeks of age (SD = 2.25), and infants in the disproportional group began treatment at 9.74 weeks (SD = 2.52). CRO infants were older at treatment initiation, with the asymmetrical group starting at 22.29 weeks of age (SD = 3.97) and the disproportional group starting at 22.16 weeks (SD = 4.95). A comparison of the starting treatment age was found to be statistically significant between the RT and CRO groups for both the asymmetrical (*p* = 0.0002*) and disproportional (*p* = 0.0008*) groups. The overall length of 2D RT treatment was, on average, 21.88 weeks (SD = 12.54) for the asymmetrical group and 27.12 months (SD = 14.45) for the disproportional group. Comparatively, 2D CRO treatment duration was found to be less for both the asymmetrical and disproportional groups, with an average duration of 20.39 weeks (SD = 6.62) and 23.14 weeks (SD = 5.03), respectively; however, these findings did not demonstrate statistical significance (*p* = 0.9391 for asymmetrical head shapes and *p* = 0.8706 for disproportionate head shapes).

Initial and final 2D measurements were assessed between the RT and CRO groups for each head shape group. On average, asymmetrical RT infants began treatment with a CVAI of 7.26 (SD = 1.81), while disproportional RT infants started with a CVAI of 5.70 (SD = 1.99). Infants in the CRO treatment group displayed a greater starting CVAI than the RT group, with asymmetrical subjects displaying a mean starting value of 8.10 (SD = 3.63) and disproportional subjects having a value of 7.07 (SD = 3.81). This relationship did not demonstrate statistical significance when comparing the two treatment modalities in the asymmetrical group (*p* = 0.5423) or the disproportional group (*p* = 0.4565). The mean ending CVAI for asymmetrical RT infants was 3.67 (SD = 1.86), and for disproportional RT infants was 2.42 (SD = 1.53), indicating that their ending CVAI values were, on average, lower than their starting values. CRO treatment displayed a similar trend as asymmetrical infants had an average ending CVAI of 3.66 (SD = 2.59), while disproportional infants had an ending CVAI of 2.48 (SD = 1.67). No statistical significance was found in the ending CVAI between the treatment groups when assessing asymmetrical head shapes (*p* = 0.7990) or disproportional head shapes (*p* = 0.9221).

The average starting CI for asymmetrical RT patients was 88.09% (SD = 3.63), whereas disproportional RT patients had an average starting CI of 93.27% (SD = 4.87). CRO treatment displayed higher average starting CI values, with 91.26% (SD = 4.84) for asymmetrical DHSs and 95.98% (SD = 3.93) for disproportional DHSs. Although close to the set level of significance, these comparisons were not statistically significant in the asymmetrical group (*p* = 0.0685) or the disproportional group (*p* = 0.0694). The mean ending CI for asymmetrical RT subjects was 86.51% (SD = 2.81) and for disproportional RT subjects was 90.14% (SD = 4.45), indicating that these ending values were closer to normal values than their starting values. Similarly, the average ending CI for CRO treatment subjects was lower than their starting values as asymmetrical DHSs had an average CI of 88.39% (SD = 3.20), and disproportional DHSs had an average CI of 90.77% (SD = 2.20). No statistical significance was found between the treatment groups for asymmetrical treatment (*p* = 0.1018) or disproportional treatment (*p* = 0.2610).

**Figure 3:**
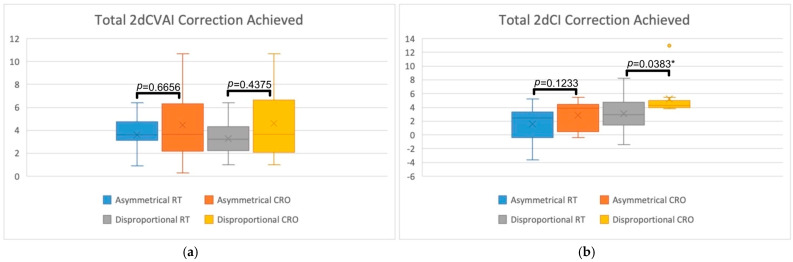
Box plots representing the total amount of 2D correction achieved when comparing RT and CRO treatments for asymmetrical and disproportional head shapes. (**a**) Visually demonstrates total 2dCVAI correction while (**b**) demonstrates total 2dCI correction. Statistical comparisons are also displayed, comparing RT to CRO treatments for each of the head shape groups, with “*” indicating statistical significance.

**Figure 4:**
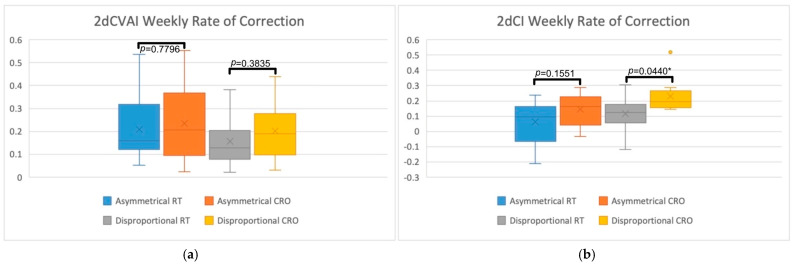
Box plots representing the 2D weekly rate of correction achieved when comparing RT and CRO treatment for asymmetrical and disproportional head shapes. (**a**) Visually demonstrates the rate of 2dCVAI correction while (**b**) demonstrates the rate of 2dCI correction. Statistical comparisons are also displayed, comparing RT to CRO treatments for each of the head shape groups, with “*” indicating statistical significance.

The total and weekly rate of 2D correction was assessed by comparing CVAI and CI measurements between the RT and CRO treatment groups for asymmetrical and disproportional head shapes. In addition to Table 3, these comparisons can be visually seen in Figure 3 and Figure 4. Figure 3 displays box plots for the total amount of 2dCVAI (3a) and 2dCI (3b) correction and also includes the comparison *p*-values for each of the treatment groups. Figure 4 displays box plots for the weekly rate of correction of 2dCVAI (4a) and 2dCI (4b), with comparison *p*-values displayed. On average, infants with asymmetry in the RT group experienced a 2dCVAI reduction of 3.59 (SD = 1.57), while disproportional RT infants experienced a 2dCVAI reduction of 3.28 (SD = 1.53). With regards to CRO treatment, the total amount of 2dCVAI correction was greater for both asymmetrical and disproportional head shapes, with the averages being 4.44 (SD = 2.99) and 4.59 (SD = 3.08), respectively. There was no statistical significance found in the asymmetrical group (*p* = 0.6656) or the disproportional group (*p* = 0.4375) when comparing RT to CRO treatments for the total amount of 2dCVAI correction achieved. Analyzing weekly 2dCVAI rate of change specifically showed that asymmetrical RT patients had a mean reduction rate of 0.21 (SD = 0.15) while disproportional RT patients displayed a mean reduction rate of 0.16 (SD = 0.1). CRO treatment demonstrated greater weekly 2dCVAI rates of change, with asymmetrical patients having an average reduction of 0.23 (SD = 0.17) and disproportional patients having an average reduction of 0.2 (SD = 0.12). Statistical analysis of the weekly rate of 2dCVAI correction between treatment modalities revealed no statistical significance in the asymmetrical group (*p* = 0.7796) or the disproportional group (*p* = 0.3835).

Similar trends for total and weekly correction were observed for 2dCI measurements. The mean total 2dCI reduction in asymmetrical RT infants was found to be 1.58 (SD = 2.47), while disproportional RT infants had a mean reduction of 3.13 (SD = 2.57). The total mean reduction in 2dCI was calculated as 2.86 (SD = 2.05) for asymmetrical CRO infants, whereas disproportional CRO infants had a mean reduction of 5.21 (SD = 2.78), indicating that a greater total amount of correction was achieved with the CRO treatment. Statistical significance was not found in the asymmetrical group when assessing total 2dCI correction (*p* = 0.1233); however, the disproportional group did display statistical significance (*p* = 0.0383*). As for the weekly rate of 2dCI change, asymmetric RT infants had a mean reduction rate of 0.06% (SD = 0.14) per week, and disproportional RT infants had a mean reduction rate of 0.12% (SD = 0.11) per week. In comparison, CRO treatment once again had greater weekly rates of 2dCI correction, with asymmetrical and disproportional infants having a mean rate of reduction of 0.14% (SD = 0.1) and 0.23% (SD = 0.11) per week, respectively. Statistical significance was found in the disproportional group (*p* = 0.0440*) but was not found in the asymmetrical group (*p* = 0.1551).

Following 2D statistical analyses, 3D demographics and treatment outcomes were assessed. Table 4 displays demographics such as the corrected treatment initiation age and the duration of 3D treatment. The 3D treatment outcomes, such as the total and weekly rate of 3dCVAI and 3dCI correction, can also be found in this table. For each of the demographics, means and standard deviations can be found, as well as comparison *p*-values and effect sizes. On average, infants undergoing RT underwent their initial 3dMD imaging at 10.33 weeks (SD = 2.13) for the asymmetrical group and 10.31 weeks (SD = 2.37) for the disproportional group. The initial 3dMD images were taken for infants undergoing CRO treatment at an older age than the RT group, being on average 23.40 weeks (SD = 4.08) for asymmetrical patients and 23.53 weeks (SD = 5.49) for disproportional patients. For both asymmetrical and disproportional DHSs, the differing starting ages for the RT and CRO groups were found to be statistically significant (asymmetrical head shapes, *p* = 0.0002*; disproportionate head shapes, *p* = 0.0008*). The average time between initial and final 3dMD imaging for asymmetrical RT infants was 21.2 weeks (SD = 11.86), while for disproportional RT infants, it was 26.35 weeks (SD = 13.84). The time between 3dMD imaging in the CRO group was found to be shorter, on average, as asymmetrical infants had a treatment time of 19.3 weeks (SD = 6.71) and disproportional infants had a treatment time of 21.8 weeks (SD = 5.52). No statistical significance was found regarding 3D treatment duration in either of the DHS groups (*p* = 1.0000 for asymmetry and *p* = 0.6725 for disproportion). The durations between initial and final 2D and 3D measurements were compared for each treatment and head shape group. Statistical significance was not found between measurement timeframes for the asymmetrical RT group (*p* = 0.2656) but was found for asymmetrical CRO (*p* = 0.0078*), disproportional RT (*p* = 0.0488*), and disproportional CRO (*p* = 0.0313*) groups.

**Figure 5:**
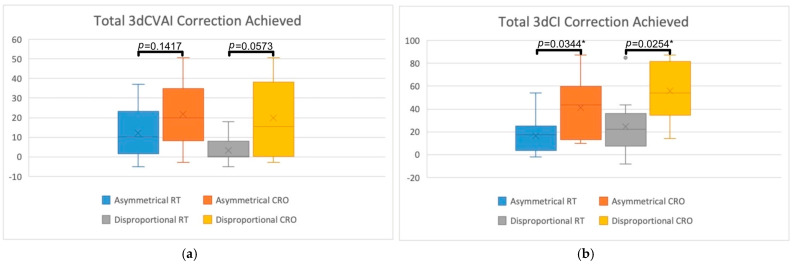
Box plots representing the total amount of 3D correction achieved when comparing RT and CRO treatments for asymmetrical and disproportional head shapes. (**a**) Visually demonstrates total 3dCVAI correction while (**b**) demonstrates total 3dCI correction. Statistical comparisons are also displayed, comparing RT to CRO treatments for each of the head shape groups, with “*” indicating statistical significance.

**Figure 6:**
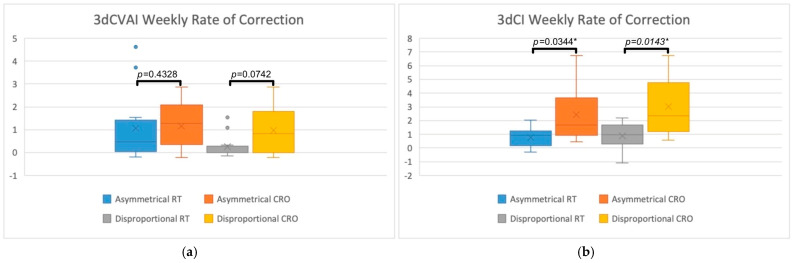
Box plots representing the 3D weekly rate of correction achieved when comparing RT and CRO treatments for asymmetrical and disproportional head shapes. (**a**) Visually demonstrates the rate of 3dCVAI correction while (**b**) demonstrates the rate of 3dCI correction. Statistical comparisons are also displayed, comparing RT to CRO treatments for each of the head shape groups, with “*” indicating statistical significance.

3D treatment outcomes were assessed by comparing 3D measurements (3dCVAI and 3dCI) between the RT and CRO groups for each head shape group. These values are visually represented in Figure 5 and Figure 6. Figure 5 displays the box plots for the total amount of 3dCVAI (5a) and 3dCI (5b) correction achieved, along with their comparison *p*-values. Figure 6 displays the box plots for the weekly rate of 3dCVAI (6a) and 3dCI (6b) correction, with statistical comparisons between the two treatment groups shown. The mean total amount of 3dCVAI correction obtained in the RT treatment group was 12.17 (SD = 13.02) for the asymmetrical group and 3.37 (SD = 6.67) for the disproportional group. The average total 3dCVAI correction was greater in the CRO treatment group when compared to RT, with 21.72 (SD = 15.36) for asymmetry and 19.97 (SD = 19.57) for disproportion. Statistical significance was nearly reached when comparing the treatment modalities for the asymmetrical (*p* = 0.1417) and disproportional groups (*p* = 0.0573). The average weekly rate of 3dCVAI correction in RT infants was 1.05 (SD = 1.55) for asymmetrical infants and 0.25 (SD = 0.52) for disproportional infants. Compared to RT treatment, CRO treatment displayed a greater average 3dCVAI, with a rate of 1.17 (SD = 0.95) in the asymmetry group and 0.98 (SD = 1.04) in the disproportion group. Comparing the two treatment groups did not reveal statistical significance in either the asymmetrical or disproportional groups (*p* = 0.4328 and *p* = 0.0742, respectively).

The mean total amount of 3dCI correction for asymmetrical RT infants was 16.71 (SD = 15.28), while for disproportional RT infants, it was 24.53 (SD = 24.01). The average total amount of 3dCI correction for CRO treatment was greater than for RT, with the asymmetrical group achieving 41.38 (SD = 27.25) and the disproportional group achieving 55.98 (SD = 25.77). Statistical significance was found for both asymmetry (*p* = 0.0344*) and disproportion (*p* = 0.0254*) when comparing treatment types. The weekly rate of 3dCI correction for the RT group was, on average, 0.77 (SD = 0.67) for asymmetry and 0.87 (SD = 0.91) for disproportion. For CRO treatment infants, the weekly rate of 3dCI correction was found to be greater versus RT, as asymmetrical infants achieved a rate of 2.42 (SD = 2.06) and disproportional infants achieved a rate of 3.02 (SD = 2.16). These rates were statistically significant in the asymmetrical group (*p* = 0.0344*) and the disproportional group (*p* = 0.0143*).

### Summary of Clinically Relevant Results

The treatment goal for asymmetric DHSs is to reduce the CVAI. In the RT group, asymmetric infants had a mean 2dCVAI reduction of 3.59 ± 1.57 and 3dCVAI reduction of 12.17 ± 13.02. Infants in the CRO treatment group had a greater mean 2dCVAI reduction (4.44 ± 2.99) and 3dCVAI reduction (21.72 ± 15.36), which was not found to be statistically significant (2d *p* = 0.6656; 3d *p* = 0.1417).

Treatment for infants with disproportionate DHSs focuses on CI reduction. Disproportionate infants in the RT group averaged a 2dCI reduction of 3.13% ± 2.57% and 3dCI reduction of 24.53 ± 24.01 while those in the CRO group had a greater average 2dCI reduction of 5.21% ± 2.78% and 3dCI reduction of 55.98 ± 25.77; RT and CRO groups were statistically different (2d *p* = 0.0383*; 3d *p* = 0.0254*).

When examining the weekly rate of change, infants with asymmetrical DHSs in the RT group had a mean 2dCVAI weekly change of 0.21 ± 0.15, while those in the CRO averaged 0.23 ± 0.17 (*p* = 0.7796). The rate of 3dCVAI weekly change was 1.05 ± 1.55 in the RT group versus 1.17 ± 0.95 in the CRO group (*p* = 0.4328).

Infants with disproportionate DHSs were found to have a greater weekly correction in the CRO group than in the RT group, which was statistically significant. The RT group had an average 2dCI weekly change of 0.12 ± 0.11, while the CRO group had an average weekly change of 0.23 ± 0.11 (*p* = 0.0440*). The mean weekly 3dCI change for infants with disproportionate DHS was 0.87 ± 0.91 in the RT group and 3.02 ± 2.16 in the CRO group (*p* = 0.0143*).

## 4. Discussion

Analyzing the results comparing the age at treatment initiation between the treatment groups showed that both 2D and 3D measurements revealed statistically significant findings. When focusing on 2D treatment initiation age in weeks, the means were 9.67 weeks for asymmetrical RT, 9.74 weeks for disproportional RT, 22.29 weeks for asymmetrical CRO, and 22.16 weeks for disproportional CRO infants. These values demonstrate that, on average, infants who underwent CRO treatment in this study started treatment at an older age relative to infants who underwent RT, regardless of head shape. The statistical significances found in the asymmetrical (*p* = 0.0002*) and disproportional (*p* = 0.0008*) groups further support the notion that of the two treatment groups, CRO patients started treatment at a later age. Very similar findings were observed for average 3dMD imaging comparisons, with starting ages of 10.33 and 10.31 weeks for asymmetrical and disproportional RT patients, respectively, and ages of 23.40 and 23.54 weeks for asymmetrical and disproportional CRO patients, respectively. Like 2D initiation age, statistical significance was found for both head shape groups regarding 3D imaging starting age (*p* = 0.0002* for asymmetry and *p* = 0.0008* for disproportion). The older age at treatment initiation for both 2D and 3D CRO treatment reflects the parameters of the study since all infants were required to enroll in the RT treatment group at two to three months of age, but the CRO group started treatment at four, five, or six months of age. These findings mirror real-world clinical scenarios, as many insurance companies will require a minimum of two months of RT before commencing CRO treatment, and RT is generally more effective in infants under 4 months of age [10]. This timing would, however, put the CRO group at a disadvantage when analyzing the weekly change rate in cranial measurements since older infants have lower growth potential and generally have slower growth [22].

The timing between 2D and 3D measurements in the different treatment modalities did not produce statistically significant results (*p* = 0.9391 for 2D asymmetry, *p* = 0.8706 for 2D disproportion, *p* = 1.0000 for 3D asymmetry, and *p* = 0.6725 for 3D disproportion), though 3d measurements were sometimes taken on different days when the subjects were not able to get their scans taken on the same day as the orthotist visit for 2D measurements. On average, 2D treatment duration was shorter in the CRO group than in the RT group (21.88 weeks for asymmetrical RT, 27.12 weeks for disproportional RT, 20.39 weeks for asymmetrical CRO treatment, and 23.14 weeks for disproportional CRO treatment). When comparing the timing of 3dMD images, infants averaged 21.20 weeks in the asymmetrical RT group, 26.35 weeks in the disproportional RT group, 19.30 weeks in the asymmetrical CRO group, and 21.80 weeks in the disproportional CRO group. Both 2D and 3D treatment durations were found to have shorter average treatment times when infants were enrolled in the CRO group regardless of their DHS group. This reflects previously referenced literature, which states that CRO treatment produces shorter treatment periods when compared to RT [14]. Considering how this study has established statistically significant older initiation ages for 2D and 3D CRO treatment, the fact that CRO treatment is clinically shorter than RT in both 2D and 3D further demonstrates the effectiveness of CRO treatment since older age has been implicated in poorer DHS treatment outcomes [12,15]. When comparing 2D and 3D treatment duration in each group, statistical significance was found for asymmetrical CRO, disproportional RT, and disproportional CRO (*p* = 0.0078*, *p* = 0.0488*, and *p* = 0.0313*, respectively) but not for asymmetrical RT (*p* = 0.2556). The statistical significances signify that the duration of treatment as measured by 2D and 3D techniques for a particular treatment group did not match up. This may be attributed to the fact that the study allowed for up to a three-week difference between 2D and 3D measurement dates. A suggestion for future studies is to ensure that 2D and 3D measurements are completed on the same day whenever possible to prevent this discrepancy in treatment time.

Initial 2dCVAI and 2dCI measurements were assessed between the treatment and head shape groups. The mean initial 2dCVAI values were calculated as 7.26 for asymmetrical RT, 5.70 for disproportional RT, 8.10 for asymmetrical CRO, and 7.07 for disproportional CRO. Although no statistical significance was found in asymmetrical (*p* = 0.5423) or disproportional (*p* = 0.4565) head shapes, the mean initial measurements were found to be greater in infants undergoing CRO treatment rather than RT. The mean initial 2dCI values were found to be 88.09% for asymmetrical RT, 93.27% for disproportional RT, 91.26% for asymmetrical CRO, and 95.98% for disproportional CRO. These averages were also greater in CRO infants relative to RT infants, but no statistical significance was found (*p* = 0.0685 for asymmetrical and *p* = 0.0694 for disproportional). These greater mean 2dCVAI and 2dCI values at treatment initiation for CRO infants match established clinical guidelines. Since CRO treatment is recommended for infants with more severe DHSs [15], it stands to reason that CRO infants would have more severe cranial measurements, as is clinically demonstrated in these results. The cranial measurements are also greater for the head shape group, which is the target of treatment. Since CVAI tracks asymmetrical DHSs and CI tracks disproportional DHSs, it would be expected that these measurements would be greater for their respective DHS group. Of note, the average 2dCVAI in this cohort was greater in the disproportional CRO group than in the RT group, and the average CI was greater in the asymmetrical CRO group than in the RT group. The reason for these measurements being greater in the CRO groups, even though they are not the target of treatment, can be attributed to infants with a DAB diagnosis. Since these infants have both an asymmetrical and disproportional presentation, the severity that warrants CRO treatment will result in higher cranial measurements even though CI is not necessarily targeted in asymmetrical infants, and CVAI is not necessarily targeted in disproportional infants.

Final 2dCVAI and 2dCI measurements were analyzed for each of the study groups. Mean final 2dCVAI values were found to be 3.67 for asymmetrical RT, 2.42 for disproportional RT, 3.66 for asymmetrical CRO, and 2.48 for disproportional CRO. The mean final 2DCI measurements were 86.51% for asymmetrical RT, 90.14% for disproportional RT, 88.39% for asymmetrical CRO, and 90.77% for disproportional CRO. No statistically significant results were found for any of the groups when assessing final 2dCVAI and 2dCI measurements (*p* = 0.7990 for asymmetrical CVAI, *p* = 0.9221 for disproportional CVAI, *p* = 0.1018 for asymmetrical CI, and *p* = 0.2610 for disproportional CI). However, each of the final cranial measurements was less than their respective initial measurements, indicating the effectiveness of RT and CROs as treatment options to address asymmetrical and disproportional head shapes.

Total 2D and 3D CVAI and CI corrections were assessed by comparing the total amount of correction achieved for these cranial measurements for each of the treatment groups. The average total 2dCVAI correction was 3.59 for asymmetrical RT, 3.28 for disproportional RT, 4.44 for asymmetrical CRO, and 4.59 for disproportional CRO. The average total 2DCI correction was 1.58 for asymmetrical RT, 3.13 for disproportional RT, 2.86 for asymmetrical CRO, and 5.21 for disproportional CRO. Neither of the 2dCVAI comparison tests yielded statistical significance (*p* = 0.6656 for asymmetrical and *p* = 0.4375 for disproportional). A difference in mean 2dCI correction was found to be statistically significant between treatment groups for disproportional head shapes (*p* = 0.0383*) but not for asymmetrical head shapes (*p* = 0.1233). For 3D correction, the mean total amount of 3dCVAI correction achieved was 12.17 for asymmetrical RT, 3.37 for disproportional RT, 21.72 for asymmetrical CRO, and 19.97 for disproportional CRO. For the mean amount of 3DCI correction, 16.71 was found for asymmetrical RT, 24.53 for disproportional RT, 41.38 for asymmetrical CRO, and 55.98 for disproportional CRO. Although none of the 3dCVAI comparison tests yielded statistically significant results (*p* = 0.1417 for asymmetrical and *p* = 0.0574 for disproportional), statistical significance was achieved for both asymmetrical (*p* = 0.0344*) and disproportional (*p* = 0.0254*) 3dCI reductions. For each of the averages, regardless of head shape in 2D or 3D, CRO treatment demonstrated greater total correction than RT. This was especially true of disproportional 2dCI, disproportional 3dCI, and asymmetrical 3dCI since these groups displayed significantly greater total correction. As already established, the CRO treatment group in this study has been characterized as starting treatment at an older age and having more severe cranial measurements. Despite these negative outcome factors, the CRO treatment groups in 2D and 3D displayed greater clinical correction and statistical correction, further highlighting how much more effective CRO treatment is at correcting DHSs than RT.

The weekly rate of 2D and 3D CVAI and CI correction was assessed by dividing the overall change by the timing between measurements, which represents the treatment duration. The mean rate of 2dCVAI correction was 0.21 for asymmetrical RT, 0.16 for disproportional RT, 0.23 for asymmetrical CRO, and 0.20 for disproportional CRO. The mean rate of 2dCI correction was 0.06 for asymmetrical RT, 0.12 for disproportional RT, 0.14 for asymmetrical CRO, and 0.23 for disproportional CRO. The analyses revealed that only the rate of disproportional 2dCI correction was statistically significant (*p* = 0.0440*), while the rest of the comparisons were not significant (*p* = 0.7796 for rate of asymmetrical 2DCVAI, *p* = 0.4375 for rate of disproportional 2DCVAI, and *p* = 0.1551 for rate of asymmetrical 2DCI). For 3D weekly correction, the mean rate of 3dCVAI correction was 1.05 for asymmetrical RT, 0.25 for disproportional RT, 1.17 for asymmetrical CRO, and 0.98 for disproportional CRO. The mean rate of 3dCI correction was 0.77 for asymmetrical RT, 0.87 for disproportional RT, 2.42 for asymmetrical CRO, and 3.02 for disproportional CRO. Analyses of the 3D rates of correction revealed that the rate of asymmetrical and disproportional 3dCVAI correction was not statistically significant (*p* = 0.4328 and *p* = 0.0742, respectively), but 3DCI correction for the asymmetrical and disproportional groups were statistically significant (*p* = 0.0344* and *p* = 0.0143*, respectively). Like the total amount of correction, the mean rates of 2D and 3D correction were found to be greater in the CRO treatment group for each of the measurements and head shapes. The greater rates of correction are likely due to CRO treatment being characterized in this study as achieving greater total correction and having shorter treatment durations. Each of the mean rates for 2D and 3D asymmetric and disproportional head shapes demonstrates this, especially the rates of disproportional 2dCI correction, asymmetrical 3dCI correction, and disproportional 3dCI correction due to their statistical significance.

There are limitations in this study that are important to note. The first is the limited number of infants enrolled in the study. To increase the sample size and curb this limitation, subjects were grouped by their type of DHS (asymmetrical or disproportional) instead of by their specific DHS diagnosis. A total of 34 infants were enrolled in this study; however, by grouping infants as asymmetrical/disproportional, the effective number of subjects increased to 48. The reason for this supposed jump in subject numbers is because DAB is both an asymmetrical and disproportional head shape. Infants with DAB undergo treatment for both cranial measurements, which warrants their inclusion in both head shape groups. Doing so allowed the sample size to be slightly larger while more broadly investigating DHSs. Even though this did generate some statistically significant results, many comparison tests did not yield statistically significant results. The reason for this is likely that there is still not a large enough subject pool to generate statistical significance, as the many 2D studies and the few 3D studies in the literature support the mean trends that were observed in this study. Future versions of this study should focus on including more infants to combat this limitation.

The second limitation of this study design is that the cut-off point for all infants, whether they achieved correction or not, was 12 months of age. This age cut-off may have potentially prevented further CRO correction from being obtained, which could have influenced the total and rates of corrections in both 2D and 3D. Future studies should attempt to follow infants up until discharge from treatment, as some of these infants did continue to wear their CRO beyond the study timeline. Doing so may maximize the opportunity for cranial correction and could have significant effects on the results.

Lastly, treatment compliance may have negatively affected the amount of correction infants achieved in this study. All infants were assumed to have adhered to CRO wear time guidelines (i.e., 23 h per day) and were only assessed based on clinical presentation. Infants who did not adhere to these guidelines may not have achieved as much correction as they would have if they were full-time CRO wearers. The use of wear-time monitoring sensors in the future could help address this limitation as it would provide an exact number of how long infants are wearing their devices and can make results easier to interpret.

The importance of interdisciplinary care should be emphasized. Although not described in this study, the study team included an imaging specialist, multiple orthotists trained in cranial remolding and repositioning therapy, a biostatistician, a pediatric neurosurgeon, and two physical therapists. Patients were referred to the treatment clinic for potential study inclusion by their pediatrician. Treatment for DHSs often involves multiple specialties, including pediatricians, physical therapists, orthotists, and caretakers. To ensure an infant’s diagnosis is handled effectively, measures must be taken to ensure the patient and caretakers are at the forefront of the treatment plan. Communication between the different specialties is key, and there must be clear communication revolving around the clinical questions and the different recommendations for treatment [29]. The roles of the different specialties should be clearly stated, and all team members must understand what is required of them. The clinicians and healthcare professionals involved with treating an infant with a DHS must adopt a multidisciplinary approach where all disciplines are included in the decision-making when developing a treatment plan to provide a more holistic form of care [30]. Caretakers should fully understand all instructions given by the healthcare professionals but are also encouraged to seek guidance from outside sources. This can come in the form of online support groups, educational workshops, parent training programs, and any educational resources that may have information on the handling of their infant’s diagnosis [30]. Promoting this team-based approach to an infant’s treatment plan will lead to improved clinical outcomes and will enhance each team member’s ability to effectively contribute to the betterment of an infant’s diagnosis. Infants included in this study were screened by the study physical therapists for torticollis at 2 months of age. If they were found to have torticollis, they were provided consecutive physical therapy treatment and a home stretching regimen until the torticollis resolved. The 3dMD imaging was overseen by our imaging specialist. RT and CRO treatments were overseen by orthotists.

The potential applications of this study can help guide the development of clinical guidelines for treating infants with DHSs. Since 2D treatment considerations have been extensively researched, the 2D results of this study only add to what is already known. The biggest applications exist in the consideration of 3D cranial correction, an area of study that has been scarcely researched. The 3D results of the study generated both clinically and statistically significant findings. These results mirror what has already been found for 2D treatment. These clinically significant findings include patients experiencing a greater rate and total amount of correction in both 3dCVAI and 3dCI when undergoing CRO treatment rather than RT. The 3D results are promising but warrant further investigation.

## 5. Conclusions

Statistical analysis revealed that in this study, infants undergoing CRO treatment started treatment at an older age and had more severe cranial measurements than infants undergoing RT. Despite the disadvantage of starting treatment at an older age, infants undergoing CRO treatment had a greater overall reduction in their DHS compared to those undergoing RT. The CRO group also completed treatment faster and achieved greater total and weekly corrections. Many of these findings may be clinically significant, and statistical significance was found for starting age, regardless of head shape, and for disproportional 2dCI total and weekly correction. The 3D statistical analysis demonstrated that, like analyses in 2D, CRO treatment has better clinical results for total and rate of 3D correction regardless of head shape when compared to RT. Of these clinical findings, some were statistically significant, including asymmetrical total and weekly 3dCI correction and disproportional total and weekly 3dCI correction. Further investigation is needed to increase the likelihood of achieving more statistically significant results by increasing the number of infants enrolled in the study, increasing the length of study enrollment, and incorporating technologies that can monitor treatment adherence. All asymmetrical head shapes saw improvement in 2dCVAI through RT and CRO treatments. All participants in the CRO group who had disproportionate head shapes saw an improvement in their 2dCI and 3dCI.

## Figures and Tables

**Table 1:** Asymmetrical and disproportional measurements characterizing each level of severity fused to classify DHS presentations. Asymmetrical classifications were based on the Children’s Healthcare of Atlanta (CHOA) severity scale, while disproportional classifications were set in this study.

Level of Severity	Asymmetry Measurement	Disproportion Measurement
Mild	3.5 ≤ CVAI < 6.5	90% > CI
Moderate	6.5 ≤ CVAI < 8.75	90% ≤ CI < 95%
Severe	8.75 ≤ CVAI < 11	95% ≤ CI < 100%
Very Severe	11 ≤ CVAI	100% ≤ CI

**Table 2:** Formulas for calculating each of the 2D and 3D cranial measurements collected. *Diagonal A* and *Diagonal B* represent the length measurements of the cranial diagonals, which were taken 30 degrees from the midline.

Cranial Measurement	Formula
2dCVAI	$\frac{\left|Diagonal\;A-Diagonal\;B\right|}{Diagonal\;A\;or\;Diagonal\;B\;\left(whichever\;is\;largest\right)}$ × 100
2dCI	$\frac{cranial\;width}{cranial\;length}$ × 100
3dCVAI	$\frac{net\;growth\;in\;flattened\;quadrants-net\;growth\;in\;bossed\;quadrants\;}{net\;growth\;in\;bossed\;quadrants\;}\times 100$
3dCI	$\frac{net\;growth\;in\;anterior\;and\;posterior\;quadrants-net\;growth\;in\;left\;and\;right\;qudrants}{net\;growth\;in\;anterior\;and\;posterior\;quadrants}$ × 100

**Table 3:** The 2D demographics, measurements, and statistical analyses of the asymmetrical and disproportional groups comparing the RT and CRO treatment groups. Comparison *p*-values with “*” indicate that statistical significance has been found, “SD” represents the standard deviations, and “ES” represents the effect size.

Demographic	Treatment Group	Measurement	Asymmetrical	Comparison *p*-Value and Effect Size	Disproportional	Comparison *p*-Value and Effect Size
Corrected Age at Start (weeks)	RT	Mean ± SD	9.67 ± 2.25	*p* = 0.0002 * ES = −0.8431	9.74 ± 2.52	*p* = 0.0008 * ES = 0.8372
		Range	4.43 to 11.86		4.43 to 13.14	
	CRO	Mean ± SD	22.29 ± 3.97		22.16 ± 4.95	
		Range	17.57 to 28.86		16.14 to 28.86	
Gender	RT	Male/Female	8/4	*p* = 0.7015 ES = −0.0976	4/8	*p* = 0.1984 ES = 0.3651
	CRO	Male/Female	8/6		7/3	
Initial 2dCVAI	RT	Mean ± SD	7.26 ± 1.81	*p* = 0.5423 ES = −0.1211	5.70 ± 1.99	*p* = 0.4565 ES = 0.1618
		Range	5.6 to 11.5		2.5 to 9	
	CRO	Mean ± SD	8.10 ± 3.63		7.07 ± 3.81	
		Range	2.8 to 15.6		2.8 to 15.6	
Final 2dCVAI	RT	Mean ± SD	3.67 ± 1.86	*p* = 0.7790 ES = 0.0505	2.42 ± 1.53	*p* = 0.9221 ES = −0.0211
		Range	0.9 to 6.4		0.9 to 5.4	
	CRO	Mean ± SD	3.66 ± 2.59		2.48 ± 1.67	
		Range	1.1 to 10.2		0.5 to 4.9	
Initial 2dCI	RT	Mean ± SD	88.09 ± 3.62	*p* = 0.0685 ES = −0.3733	93.27 ± 4.87	*p* = 0.0694 ES = 0.4079
		Range	83.4 to 93.2		86.8 to 103.2	
	CRO	Mean ± SD	91.26 ± 4.84		95.98 ± 3.93	
		Range	82.6 to 99.3		91.4 to 105.2	
Final 2dCI	RT	Mean ± SD	86.51 ± 2.81	*p* = 0.1018 ES = −0.3332	90.14 ± 4.45	*p* = 0.2610 ES = 0.2463
		Range	82.00 to 90.2		84.1 to 100.4	
	CRO	Mean ± SD	88.39 ± 3.20		90.77 ± 2.20	
		Range	82.9 to 94.8		87.6 to 94.8	
2D Treatment Time (weeks)	RT	Mean ± SD	21.88 ± 12.54	*p* = 0.9391 ES = −0.0151	27.12 ± 14.45	*p* = 0.8706 ES = −0.0352
		Range	6.71 to 43.14		8.14 to 46	
	CRO	Mean ± SD	20.39 ± 6.62		23.14 ± 5.03	
		Range	10.86 to 31.29		15 to 31.29	
Total Correction Achieved (2dCVAI)	RT	Mean ± SD	3.59 ± 1.57	*p* = 0.6656 ES = −0.0858	3.28 ± 1.53	*p* = 0.4375 ES = 0.1687
		Range	0.9 to 6.4		1 to 6.4	
	CRO	Mean ± SD	4.44 ± 2.99		4.59 ± 3.08	
		Range	0.28 to 10.7		1 to 10.7	
Total Correction Achieved (2dCI)	RT	Mean ± SD	1.58 ± 2.47	*p* = 0.1233 ES = −0.3128	3.13 ± 2.57	*p* = 0.0383 * ES = 0.4712
		Range	−3.6 to 5.2		−1.4 to 8.2	
	CRO	Mean ± SD	2.86 ± 2.05		5.21 ± 2.78	
		Range	−0.4 to 5.5		3.8 to 13	
Correction Rate per Week of Treatment (2dCVAI)	RT	Mean ± SD	0.21 ± 0.15	*p* = 0.7796 ES = 0.0555	0.16 ± 0.1	*p* = 0.3835 ES = −0.1898
		Range	0.05 to 0.54		0.02 to 0.38	
	CRO	Mean ± SD	0.23 ± 0.17		0.2 ± 0.12	
		Range	0.02 to 0.55		0.03 to 0.44	
Correction Rate per Week of Treatment (2dCI)	RT	Mean ± SD	0.06 ± 0.14	*p* = 0.1551 ES = 0.2875	0.12 ± 0.11	*p* = 0.0440 * ES = −0.4569
		Range	−0.21 to 0.24		−0.12 to 0.30	
	CRO	Mean ± SD	0.14 ± 0.1		0.23 ± 0.11	
		Range	−0.03 to 0.29		0.14 to 0.52	

**Table 4:** The 3D demographics, measurements, and statistical analyses of the asymmetrical and disproportional groups comparing RT and CRO treatment groups. Comparison *p*-values, with “*” indicating that statistical significance has been found. “SD” represents the standard deviations and “ES” represents the effect size.

Demographic	Treatment Group	Measurement	Asymmetrical	Comparison *p*-Value and Effect Size	Disproportional	Comparison *p*-Value and Effect Size
Corrected Age at Start (weeks)	RT	Mean ± SD	10.33 ± 2.13	*p* = 0.0002 * ES = −0.8428	10.31 ± 2.37	*p* = 0.0008 * ES = 0.8374
		Range	5.57 to 12.71		5.57 to 14.14	
	CRO	Mean ± SD	23.40 ± 4.08		23.54 ± 5.49	
		Range	19.43 to 30.86		16.14 to 30.86	
3D Treatment Time (weeks)	RT	Mean ± SD	21.2 ± 11.86	*p* = 1.0000 ES = 0	26.35 ± 13.84	*p* = 0.6725 ES = −0.0914
		Range	6.29 to 41.29		7.57 to 45.00	
	CRO	Mean ± SD	19.3 ± 6.71		21.8 ± 5.52	
		Range	10.86 to 31.00		12.71 to 29.29	
Percent Change in 3dCVAI	RT	Mean ± SD	12.17 ± 13.02	*p* = 0.1417 ES = −0.2976	3.37 ± 6.67	*p* = 0.0573 ES = 0.4288
		Range	−5.09 to 37.10		−5.09 to 18.00	
	CRO	Mean ± SD	21.72 ± 15.36		19.97 ± 19.57	
		Range	−2.73 to 50.55		−2.73 to 50.55	
Percent Change in 3dCI	RT	Mean ± SD	16.71 ± 15.28	*p* = 0.0344 * ES = −0.4388	24.53 ± 24.01	*p* = 0.0254 * ES = 0.5131
		Range	−2.00 to 53.97		−8.36 to 84.71	
	CRO	Mean ± SD	41.38 ± 27.25		55.98 ± 25.77	
		Range	9.98 to 87.44		14.18 to 87.44	
Correction Rate per Week of Treatment (3dCVAI)	RT	Mean ± SD	1.05 ± 1.55	*p* = 0.4328 ES = −0.1563	0.25 ± 0.52	*p* = 0.0742 ES = 0.4006
		Range	−0.20 to 4.62		−0.14 to 1.54	
	CRO	Mean ± SD	1.17 ± 0.95		0.98 ± 1.04	
		Range	−0.21 to 2.87		−0.21 to 2.87	
Correction Rate per Week of Treatment (3dCI)	RT	Mean ± SD	0.77 ± 0.67	*p* = 0.0344 * ES = −0.4388	0.87 ± 0.91	*p* = 0.0143 * ES = 0.5694
		Range	−0.32 to 2.03		−1.10 to 2.19	
	CRO	Mean ± SD	2.42 ± 2.06		3.02 ± 2.16	
		Range	0.47 to 6.73		0.55 to 6.73	

## Data Availability

The University of Texas Southwestern Medical Center requires participants to be notified in the informed consent form if a participant’s individual data will be shared through a controlled or open-access repository and the risks and benefits associated with each sharing method. When this study was performed, the informed consent form did not include a data-sharing description; therefore, at this time, we cannot publicly share data at the participant level. Data may be requested through a data use agreement by contacting Tiffany.Graham@utsouthwestern.edu.
